# O6.5. LINKING CORTICAL AND CONNECTIONAL PATHOLOGY IN SCHIZOPHRENIA

**DOI:** 10.1093/schbul/sby015.226

**Published:** 2018-04-01

**Authors:** Maria Di Biase, Vanessa Cropley, Luca Cocchi, Alexander Fornito, Fernando Calamante, Eleni Ganella, Christos Pantelis, Andrew Zalesky

**Affiliations:** 1 Melbourne Neuropsychiatry Centre; 2 The University of Melbourne, Melbourne Neuropsychiatry Centre; 3 QIMR Berghofer Medical Research Institute; 4 School of Psychology and Psychiatry & Monash Biomedical Imaging, Monash University; 5 University of Melbourne

## Abstract

**Background:**

Schizophrenia is associated with cortical thinning and breakdown in white matter microstructure. Whether these pathological processes are related remains unclear. We used multimodal neuroimaging to investigate the relation between regional cortical thinning and breakdown in adjacent infracortical white matter as a function of age and illness duration.

**Methods:**

Structural magnetic resonance and diffusion images were acquired in 218 schizophrenia patients and 167 age-matched healthy controls to map cortical thickness (CT) and fractional anisotropy (FA) in regionally adjacent infracortical white matter at various cortical depths.

**Results:**

Between-group differences in CT and infracortical FA were inversely correlated across cortical regions (r=−0.5, p<0.0001), such that the most anisotropic infracortical white matter was found adjacent to regions with extensive cortical thinning. This pattern was evident in early (20 years: r=−0.3, p=0.005) and middle life (30 years: r=−0.4, p=0.004, 40 years: r=−0.3, p=0.04), but not beyond 50 years (p>0.05). Frontal pathology contributed most to this pattern, with extensive cortical thinning in patients compared to controls at all ages (p<0.05); in contrast to initially increased frontal infracortical FA in patients at 30 years, followed by rapid decline in frontal FA with age (rate of annual decline; patients: 0.0012, controls 0.0006, p<0.001).

**Discussion:**

Cortical thinning and breakdown in white matter anisotropy are inversely related in young schizophrenia patients, with abnormally elevated white matter myelination found adjacent to frontal regions with extensive cortical thinning. We argue that elevated frontal anisotropy reflects regionally-specific, compensatory responses to cortical thinning, which are eventually overwhelmed with increasing illness duration.

